# Chromosomal Microarray Analysis of Consecutive Individuals with Autism Spectrum Disorders Using an Ultra-High Resolution Chromosomal Microarray Optimized for Neurodevelopmental Disorders

**DOI:** 10.3390/ijms17122070

**Published:** 2016-12-09

**Authors:** Karen S. Ho, E. Robert Wassman, Adrianne L. Baxter, Charles H. Hensel, Megan M. Martin, Aparna Prasad, Hope Twede, Rena J. Vanzo, Merlin G. Butler

**Affiliations:** 1 Lineagen, Inc., Salt Lake City, UT 84109, USA; bwassman@lineagen.com (E.R.W.); abaxter@lineagen.com (A.L.B.); chensel@lineagen.com (C.H.H.); mmartin@lineagen.com (M.M.M.); aprasad@lineagen.com (A.P.); htwede@lineagen.com (H.T.); rvanzo@lineagen.com (R.J.V.); 2 Department of Pediatrics, University of Utah, Salt Lake City, UT 84132, USA; 3 Departments of Psychiatry, Behavioral Sciences and Pediatrics, University of Kansas Medical Center, Kansas City, UT 66160, USA; mbutler4@kumc.edu

**Keywords:** chromosomal microarray, copy number variants, neurodevelopmental disorders, autism spectrum disorder, variants of unknown significance, FirstStepDx PLUS

## Abstract

Copy number variants (CNVs) detected by chromosomal microarray analysis (CMA) significantly contribute to understanding the etiology of autism spectrum disorder (ASD) and other related conditions. In recognition of the value of CMA testing and its impact on medical management, CMA is in medical guidelines as a first-tier test in the evaluation of children with these disorders. As CMA becomes adopted into routine care for these patients, it becomes increasingly important to report these clinical findings. This study summarizes the results of over 4 years of CMA testing by a CLIA-certified clinical testing laboratory. Using a 2.8 million probe microarray optimized for the detection of CNVs associated with neurodevelopmental disorders, we report an overall CNV detection rate of 28.1% in 10,351 consecutive patients, which rises to nearly 33% in cases without ASD, with only developmental delay/intellectual disability (DD/ID) and/or multiple congenital anomalies (MCA). The overall detection rate for individuals with ASD is also significant at 24.4%. The detection rate and pathogenic yield of CMA vary significantly with the indications for testing, age, and gender, as well as the specialty of the ordering doctor. We note discrete differences in the most common recurrent CNVs found in individuals with or without a diagnosis of ASD.

## 1. Introduction

Neurodevelopmental disabilities, including developmental delay (DD), intellectual disability (ID), and autism spectrum disorder (ASD) affect up to 15% of children [1]. However, in the vast majority of cases, a child’s clinical presentation does not allow for a definitive etiological diagnosis.

Autism spectrum disorder is characterized by impairment in three domains with onset of one or more of these before the age of 3 years: social interaction; communication skills; and restricted, repetitive, and stereotyped patterns of behavior, interests, and activities. About 40% of individuals with ASD also have a learning disability, and roughly 30% have other co-morbidities such as seizures [1,2,3].

The etiology of ASD is complex and prominently involves genetic factors, including single gene changes, large genomic structural changes (i.e., deletions or duplications) known as copy number variants (CNV), and other polygenic conditions often influenced by the environment and epigenetic changes [2,3]. Genetic testing to pinpoint the underlying cause of ASD is critical to an individual’s clinical management. Further, chromosomal microarray analysis (CMA) has demonstrated the highest diagnostic yield for individuals with ASD as compared to other genetic tests. Therefore, along with previously recognized indications of DD, ID and multiple congenital anomalies (MCA), children and adults presenting with ASD should be offered CMA as a first tier genetic evaluation based on the clinical guidelines from multiple professional societies [2,3,4,5,6,7,8,9,10].

Microarrays that employ a variety of designs and range of coverage for certain genomic regions have been applied to the clinical testing of individuals with these conditions. Diagnostic yield has increased over time as such arrays have evolved to include better coverage [7,8,9,10,11,12,13,14,15,16,17,18,19,20,21]. In 2011 the ACMG issued a guideline regarding optimal microarray design and recommended inclusion of additional probe content in areas of known clinical relevance [22]. The following data summarizes our experience with real-world clinical CMA testing of individuals with a diagnosis of ASD in a CLIA-certified laboratory over a period of 4.2 years. The microarray platform utilized in this study was specifically designed to increase detection of CNVs in genomic regions of demonstrated relevance to DD/ID/ASD. We also compare our experience to a non-ASD population clinically tested in parallel in the same laboratory and on the same platform.

## 2. Results

A total of 10,351 custom, ultra-high resolution CMAs optimized for the detection of neurodevelopmental disorders (FirstStepDx PLUS^®^ (FSDX PLUS^®^)) were performed over a period of four years. This testing population had a M:F ratio of 2.5:1 and a mean age of 7.0 years. Based on ICD-9 and ICD-10 codes at the time of referral, 55% of cases represented patients with a diagnosis of ASD with or without other features (ASD+ and ASD only, respectively). Table 1 and Table 2 show summary data of our neurodevelopmental patient cohort.

The mean age of testing was younger for the non-ASD group versus the ASD only group or the ASD+ group (Table 2). Overall, neurologists were the most common referring physicians (36%), followed by developmental pediatricians (31%), pediatricians (16%), and medical geneticists (14%). Although psychiatrists referred only 2% of total cases, they had the highest percentage of their referrals for an indication of ASD (72%) with or without other features, while only 29% of the cases referred by geneticists had an indication including ASD. Of the total caseload, 74% of the ASD cases were referred by pediatric neurologists and developmental/behavioral pediatricians.

Overall, we observe a 28.0% diagnostic yield for potentially abnormal CNVs (Table 3), with an average of 1.2 reportable CNVs detected per individual. Interestingly, the rate of pathogenic findings is significantly lower (4.4%) when the diagnostic indication is ASD only compared to diagnostic indication of DD/ID/MCA without a reported diagnosis of ASD (non-ASD cohort) (12.5%) (*p* < 0.001). The pathogenic rate is only slightly higher in the ASD+ group (6.7%). However, VOUS rates are similar across the ASD only, ASD+, and non-ASD cohorts. The observation of lower rates of reportable CNVs for the ASD only cohort as compared to the ASD+ cohort, and the non-ASD cohort is maintained when looking at the overall yields for each group.

We stratified our cohort by age to determine whether there were differences in the rate of pathogenic findings when data were viewed this way, and found that rates were highest in the non-ASD cohort in the first year of life (18.9%), which then dropped to 10.7%–12.4% during childhood and adolescence (Table 4). In the ASD cohort, the overall pathogenic rate was slightly higher for individuals with ASD+ as compared to the overall pathogenic rate for individuals with ASD only. The pathogenic rate in the ASD+ cohort started at 4.1% in the youngest group and rose to 8.5% in the 5.5–10 years range (Table 5). The pathogenic rate in the ASD only cohort rose gradually with age from 3.4% in the youngest cohort (0–3.4 years) to a peak at 7.0% in adolescence (Table 6).

While largely targeting a pediatric population, a subset of 383 patients comprised adults over 18 years of age at the time of testing (parental and sibling studies excluded). Interestingly, in the non-ASD cohort the pathogenic rate in adults tested was 18.1%, the highest for any age cohort in this population after the first year of life (Table 4). While the percentage of pathogenic findings in adults with an indication of ASD (9.8% for ASD+; 5.6% for ASD only) was much lower than in the non-ASD population. It is worth noting that older age cohorts maintain high diagnostic yields with or without indications of ASD (Table 4, Table 5 and Table 6).

**Figure 1:**
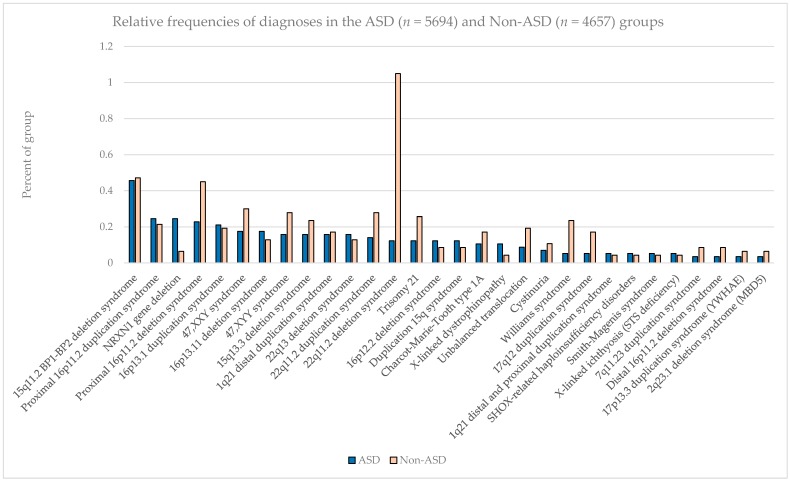
Relative frequencies of diagnoses in the combined ASD (*n* = 5694) and Non-ASD (*n* = 4657) groups.

**Figure 2:**
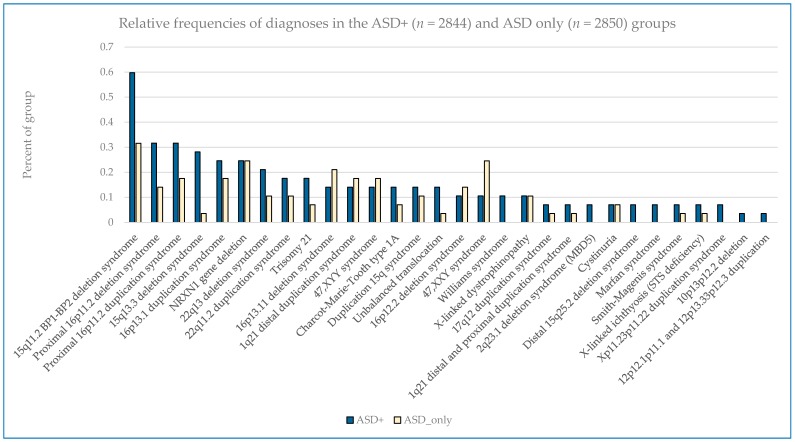
Relative frequencies of diagnoses in the ASD+ (*n* = 2844) and ASD only (*n* = 2850) groups.

**Figure 3:**
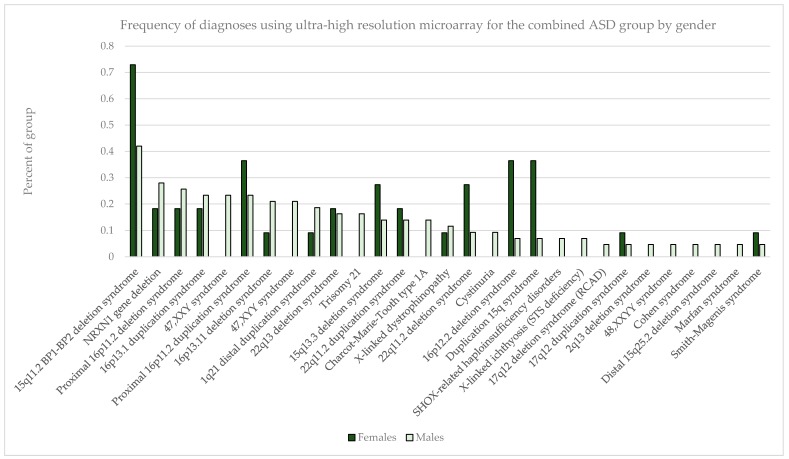
Frequency of diagnoses for combined ASD group by gender. Total females = 2929; total males = 7422.

The most common pathogenic findings detected in this series of individuals evaluated by CMA are shown in Figure 1, Figure 2 and Figure 3. We observed, in some cases, striking differences in the frequencies of pathogenic findings when patients are grouped by testing indications and/or gender. For example, the 22q11.2 deletion and, to a lesser extent, the proximal 16p11.2 deletion, were far more prevalent in the non-ASD group than in the combined ASD group, suggesting that indications other than ASD in these patients are common or that ASD is less readily diagnosed in these subgroups (Figure 1). However, the 15q11.2 BP1–BP2 deletion (also known as the Burnside-Butler susceptibility locus) and the proximal 16p11.2 duplication were equally likely to be detected if ASD was indicated or not (Figure 1). In contrast, *NRXN1* gene deletions were much more common when ASD was indicated in comparison to when it was not, by a factor of nearly 4-fold (Figure 1). Figure 2 displays some similarities and differences between the ASD+ and ASD only populations; for example the 15q11.2 BP1–BP2 deletion is the most common finding for both cohorts, but detection frequencies vary significantly for other findings, with 47,XXY being the next most common for the ASD-only cohort while the proximal 16p11.2 deletions and duplications are the next most frequent finding in the ASD+ cohort.

Males outnumbered females in our study population, and the rate of abnormality differed significantly with females having higher rates of pathogenic findings across all diagnostic groupings (*p* < 0.001 for non-ASD and ASD+ groups as well as overall); ASD only detection rates in females vs. males were not statistically different (*p* = 0.22) (Table 7). In the combined ASD group, in addition to specific sex-limited diagnoses like 47,XXY and 47,XYY, there were excesses by gender in the prevalence of several common abnormalities, notably with the 15q11.2 BP1–BP2 deletion, 15q duplication, 15q13.3 deletion, proximal 16p11.2 duplication, 16p12.2 deletion, and 22q11.2 deletion all skewed toward a female preponderance by up to 2–3-fold (Figure 3).

## 3. Discussion

CMA is the guideline-recognized first-tier test in the evaluation of individuals with DD/ID, MCA, and most recently ASD [4,6,7,8,9,10,15]. CMA yields significant rates of pathogenic or potentially pathogenic (VOUS) results [2,11,12,13,14,15,16,17,18,19,20], which have clinical utility for the case-by-case clinical management of individuals with these individually rare disorders [23,24,25,26,27,28,29,30,31,32,33,34].

Since the introduction of CMA technology, the total genomic content with probe coverage has progressively increased leading to higher diagnostic yields and better resolution of chromosomal abnormalities. Collectively this trend has resulted in corresponding increases in the clinical value of CMA testing [11,12,13,14,15,16,17,18,19,20,21,24,25,26,27,28,29,30,31,32,33,34]. In addition to guidelines on the clinical indications for CMA, the American College of Medical Genetics and Genomics (ACMG) has issued guidance on the appropriate content and design of such arrays and specifically opined that, “It is desirable to have enrichment of probes targeting dosage-sensitive genes known to result in phenotypes consistent with common indications for a genomic screen (e.g., intellectual disability, developmental delays, autism, and congenital anomalies)” [22]. We report here on over four years of clinical experience with a real-world referral base for testing on an ultra-high resolution chromosomal microarray specifically designed to extend the scope of detection for individuals with ASD and other neurodevelopmental disorders. This microarray was optimized through the addition of probes targeting genomic regions more recently identified to have pathogenic relevance for DD, ID, and ASD [21,35,36].

The overall detection rate in this series for clinically established pathogenic CNVs of 8.6% is comparable to other reported series/platforms [2,11,12,13,14,15,16,17,18,19,20], despite the inherent bias toward lower rates based on the real-world referral base and higher percentage of individuals with ASD in this population. When ASD is not among the testing indications, the rate of pathogenic findings is 12.5% and the overall diagnostic yield is 32.6%, both of which are at the upper end of reported diagnostic rates. We have previously shown that diagnostic yield varies significantly on a multivariate basis including but not limited to: referring physician specialty, age of patient at testing, patient gender, and referring indication or combination of indications for testing [20]. When we look in this study at the influence of an ASD diagnosis on pathogenic diagnosis rates by practitioners, geneticists have the highest detection rate when there is no indication of ASD for testing, but also the lowest rate for individuals with ASD only.

The overall detection rate in this series for clinically established pathogenic CNVs of 8.6% is comparable to other reported series/platforms [2,11,12,13,14,15,16,17,18,19,20], despite the inherent bias toward lower rates based on the real-world referral base and higher percentage of individuals with ASD in this population. When ASD is not among the testing indications, the rate of pathogenic findings is 12.5% and the overall diagnostic yield is 32.6%, both of which are at the upper end of reported diagnostic rates. We have previously shown that diagnostic yield varies significantly on a multivariate basis including but not limited to: referring physician specialty, age of patient at testing, patient gender, and referring indication or combination of indications for testing [20]. When we look in this study at the influence of an ASD diagnosis on pathogenic diagnosis rates by practitioners, geneticists have the highest detection rate when there is no indication of ASD for testing, but also the lowest rate for individuals with ASD only.

While significantly lower than both the overall population and the ASD-excluded sub-population (*p* < 0.0001), the diagnostic yield in all cases including ASD (ASD only and ASD+) are 5.4% pathogenic, 19.0% VOUS, and 24.4% overall. This overall rate exceeds those previously reported [3,14,15,16,17] and this supports the value of incremental targeted content for CMA design.

We detected VOUS at an overall rate of 19.4%. Although earlier literature did not typically consider VOUS in the diagnostic yield, this was due to inconsistent criteria for reporting, lack of established databases of normal population variants, and limited sharing of data [10]. Today, it is common and reasonable to consider VOUS in the overall diagnostic yield [3,14,15]. Many VOUS results will evolve into clearly pathogenic findings based on emerging clinical evidence [37]. The excess of males to females in our overall and combined ASD cohorts are consistent with previous reports. In addition, we confirmed the higher rates of abnormalities in the tested female populations in comparison to males, which has been previously observed [3,14].

A recent report by one of us (MGB) on CMA use in individuals with DD and ASD at a single midwest genetics center using relatively low resolution (<180 K) oligonucleotide arrays found 6 of 65 patients with ASD (9%) to have a pathogenic finding and 20% overall (13/65) had a reportable CNV. This is higher than the rate of pathogenic findings in our ASD population (6.7% ASD+; 4.4% ASD only); however, this was a much smaller total population and in addition was a closely-studied cohort within an academic medical center where all patients had complete clinical genetics evaluations [3]. The overall diagnostic yield of 20% in that study was similar to rates reported by Shen et al. [14] (18.2% in 932 patients with ASD) and Schaefer et al. [15] (21% or 14 patients 68 with ASD) but is lower than the 24.4% observed with the optimized array in this series.

Numerous studies have now demonstrated the clinical actionability and utility of CMA testing [23,24,25,26,27,28,38,39], and increased yield as described here will extend the range and scope of this utility. The increased rate of CNVs classified as VOUS is therefore of potential clinical importance in this setting and consideration of location, gene content, and other factors may help clinicians with such patients despite the complexities of interpreting them and counseling families as to their potential significance. Of critical importance is the ongoing evaluation of novel methods to assess the potential role of VOUS in the underlying pathology of individual patients. This process will allow us as a community to realize the maximum benefit of the increased detection rate achieved through array and interpretation optimization. Furthermore, VOUS results have been clearly demonstrated to be of great importance to parents of patients with DD/ID/ASD [34,40,41,42,43].

It is estimated that at least 20% of individuals with ASD have an underlying genetic syndrome, but a survey of a large autism center showed that less than 10% of their population had received any form of genetic evaluation [44]. The evidence here also supports that patients with a diagnosis of ASD remain under-tested overall. The fact that the age of CMA diagnosis in those with ASD is a full standard deviation greater than the potential age of clinical diagnosis speaks to the delay or reticence in taking critical steps to better medically manage these patients. The direct correlation between higher rate of detected abnormalities and age in the ASD cohort suggests that earlier use of CMA and perhaps other genetic testing methods may be important for early intervention.

While still a relatively small sub-cohort, it is remarkable that adults (>18 years old) tested also have the highest pathogenic CNV rate of all diagnostic groups examined. This could be reflective of severity in that particular age group. For example, clinicians/families might believe that testing isn’t as valuable for adults but perform it anyway when the individual is considered to be relatively severely impaired. In addition, this may also reflect the desire for adults (or adult siblings of the individual with clinical features) to define recurrence risk to their potential offspring.

Even before prior CMA was introduced into clinical use, the most common chromosome abnormalities associated with apparently isolated ASD were duplication of the 15q11–q13 region (typically of maternal origin) [45] and large microdeletions in the chromosome 16p11.2 and 22q regions reportedly accounting for as many as 1%–5% of ASD related abnormalities each [3,23]. Although these well-described recurrent abnormalities were prominent and relatively more abundant in our ASD cohorts, their prevalence was not as high as predicted by the literature; however, some of the reports suggesting high rates may have been biased by multiplex families.

Partial deletions involving the *NRXN1* gene are now well-described abnormalities, and impairment of the function of the synaptic adhesion protein it encodes, leading to a potential loss of synaptic integrity, is thought to be central to the pathogenesis of ASD [46,47]. *NRXN1* gene deletions were significantly over-represented in both our ASD+ and ASD only groups but were also observed at least occasionally in the non-ASD group. The latter may be due to either early testing for some other clinical feature prior to the formal recognition of ASD-related features, the indubitable inadequacies of relying on physician coding on test requisitions for phenotypic data, or true overlap into other neurodevelopmental conditions without ASD.

The observational study of a large consecutive series of genetic testing for neurodevelopmental disorders, over half of which had feature of ASD highlights the significant value of CMA in defining not merely the underlying etiology but in directing future research into the underlying pathophysiology for improved and ultimately targeted treatments. While it is not ideal to rely on ICD-9/10 coding on test requisitions to define the phenotypic sub-groups of this population and parse the results relevant to ASD, the comparability and trends of this large data set suggest that the conclusions are neither random nor merely directional, and likely reflects a reasonable picture of the scope of abnormalities in these populations. Improved diagnostic tools will lead to increased clinical utility and in the end better clinical management.

## 4. Materials and Methods

### 4.1. Patient Ascertainment

A consecutive series of 10,351 real-world samples referred for CMA to a CLIA-licensed clinical laboratory for etiological diagnosis of DD/ID/ASD and MCAs between July 2012 and September 2016 was reviewed for clinical characteristics and related diagnoses. The overwhelming majority of samples were buccal swabs, however 1037/10351 (~10%) cases were conducted on blood specimens. Testing indications were delineated based on International Classification of Diseases, Clinical Modification, Revisions 9 or 10, (ICD-9, ICD-10) (Centers for Medicare & Medicaid Services (cms.gov)).

### 4.2. Microarray Design

The FSDX PLUS^®^ microarray utilized in this study, and its analytical and clinical validation, have been described in detail elsewhere [21]. It is an expanded whole genome chromosome microarray (CMA) built upon the ultra-high resolution Affymetrix CytoscanHD^®^ platform (Santa Clara, CA, USA) plus 88,435 custom probes targeting genomic regions strongly associated with DD/ID/ASD [14,15,16,17,18,19,20,21,22,23,24]. Both copy number (CNV) and single nucleotide polymorphic (SNP) probes are included in the array, which is consistent with the ACMG guideline for CMA design, as is the “enrichment of probes targeting dosage-sensitive genes known to result in phenotypes consistent with common indications for a genomic screen” [22]. Such critical regions that did not contain >1 probe/1000 bp on the baseline array were supplemented with additional probe content to provide improved detection of smaller deletions and duplications. Additional probe enrichment was of genomic regions identified by our prior studies and elsewhere in the medical literature of published copy number variants and individual genes associated with DD/ID/ASD [23,24,25,26,27,28,29,30,31,32,33,34,35,36]. The increase in analytical sensitivity resulting from this additional 3.3% probe content has been calculated to be 2.6% [21].

### 4.3. CMA Performance and Interpretation

CMA was routinely performed on DNA extracted by standard methodologies from buccal swab samples (ORAcollect^®^) in a CLIA-certified laboratory. CMA reagents and equipment were as specified by Affymetrix. Established cytogenetic criteria for interpretation were routinely applied with a minimum of 25-consecutive impacted probes as the baseline determinant for deletions and 50 probes for duplications [35]. Rare CNVs (<1% overall population frequency) were determined to be “pathogenic” if there is sufficient published clinical evidence (at least two independent publications) to indicate that haploinsufficiency or triplosensitivity of the region or gene(s) involved is causative of clinical features. If only preliminary evidence for a causative role for the region or gene(s) therein was found, they were classified as variants of unknown significance (VOUS) as were areas of absence of heterozygosity (AOH) which may increase the risk for conditions with autosomal recessive inheritance or conditions with parent-of-origin/imprinting effects. Cases with only CNVs contained in databases such as the Database of Genomic Variants (DGV) [39] that document presumptively benign CNVs were reported as normal.

### 4.4. Statistical Methods

Chi-square tests for independence and 2-sided *t*-tests for normal distribution were applied to the data for determination of significance of findings.

## 5. Conclusions

Ultra-high resolution CMA has demonstrated great value in the clinical assessment of neurodevelopmental disorders. The diagnostic yield of the optimized CMA platform described here is dependent on many factors, including patient gender, age at testing, clinical presentation, and specialty of the ordering physician. Pathogenic findings give insights into the etiology of patients’ neurodevelopmental conditions, and in many cases positively impact medical management decisions. The development of novel and accurate methods to interpret the potential pathenogenicity of VOUS will further enable patients and their physicians to realize the maximum benefits of genetic testing for clinical care.

## Figures and Tables

**Table 1:** Summary data of our neurodevelopmental patient cohort.

Cohort Characteristics	Total
Number of Samples	10,351
Number of Males/Females (2.5:1)	7422/2929
Non-ASD *	4657
Any ASD ^†^	5694
ASD+ ^‡^	2844 (27.4%)
ASD only **^§^**	2850 (27.5%)

* “Non-ASD” represents that portion of the cohort with no testing indication of ASD; ^†^ “Any ASD” refers to the portion of the cohort that has ASD as a sole testing indication, or in combination with any other testing indications, thus it represents both “ASD only” and “ASD+” cohorts combined; ^‡^ “ASD+” refers to the portion of the cohort with an indication of ASD as well as another testing indication, such as MCA, seizures, DD, and/or ID; **^§^** “ASD only” refers to the portion of the cohort with ASD as the only testing indication.

**Table 2:** Mean age at chromosomal microarray analysis (CMA) testing, grouped by diagnostic referral codes.

Population	Mean Age at Testing (Years)	Standard Deviation (Years)
All	7.0	5.6
Non ASD *	6.5	6.0
ASD only ^†^	7.3	5.0
ASD+ ^‡^	7.5	5.1

* Non-ASD” represents that portion of the cohort with no testing indication of ASD; ^†^ “ASD only” refers to the portion of the cohort with ASD as the only testing indication; ^‡^ “ASD+” refers to the portion of the cohort with an indication of ASD as well as another testing indication, such as MCA, seizures, DD, and/or ID.

**Table 3:** Diagnostic yields of genetic testing in 10,351 consecutive children with neurodevelopmental disorders by diagnostic referral codes.

Result	All	Non-ASD *	Any ASD ^†^	ASD+ ^‡^	ASD Only ^§^
Pathogenic	8.6%	12.5%	5.4%	6.5%	4.4%
VOUS	19.4%	20.1%	19.0%	19.4%	18.5%
Overall Yield	28.1%	32.6%	24.4%	25.9%	22.9%

* “Non-ASD” represents that portion of the cohort with no testing indication of ASD; ^†^ “Any ASD” refers to the portion of the cohort that has ASD as a sole testing indication, or in combination with any other testing indications, thus it represents both “ASD only” and “ASD +” cohorts combined; ^‡^ “ASD+” refers to the portion of the cohort with an indication of ASD as well as another testing indication, such as MCA, seizures, DD, and/or ID; **^§^** “ASD only” refers to the portion of the cohort with ASD as the only testing indication.

**Table 4:** Diagnostic yield by age in patients without autism spectrum disorder (ASD) (1750 (37.6%) females, 2907 (62.4%) males, Total *n* = 4657).

Age in Years	Number of Tests	Pathogenic (% Yield)	VOUS (% Yield)	Normal (% Yield)
0–1.0	439	83 (18.9%)	84 (19.1%)	272 (62.0%)
1.0–3.5	1407	151 (10.7%)	275 (19.5%)	981 (69.7%)
3.5–5.4	688	81 (11.8%)	147 (21.4%)	460 (66.8%)
5.5–10	1107	132 (11.9%)	244 (22.0%)	731 (66.1%)
10.1–18	834	103 (12.4%)	155 (18.6%)	576 (69.0%)
18+	182	33 (18.1%)	29 (15.9%)	120 (66.0%)
Total	4657	583 (12.5%)	934 (20.1%)	3140 (67.4%)

**Table 5:** Diagnostic yield by age in patients with ASD and other indications (610 (21.4%) females, 2234 (78.6%) males, Total *n* = 2844).

Age in Years	Number of Tests	Pathogenic (% Yield)	VOUS (% Yield)	Normal (% Yield)
0–3.4 *	735	30 (4.1%)	156 (21.2%)	549 (74.7%)
3.5–5.4	630	34 (5.4%)	114 (18.1%)	482 (76.5%)
5.5–10	710	60 (8.5%)	132 (18.6%)	518 (72.9%)
10.1–18	657	49 (7.5%)	126 (19.2%)	482 (73.3%)
18+	112	11 (9.8%)	24 (21.4%)	77 (68.8%)
Total	2844	184 (6.5%)	552 (19.4%)	2108 (74.1%)

* Due to the typical age of clinical recognition and diagnoses for ASD, the range of 0–3.4 years was used for the youngest grouping in this Table.

**Table 6:** Diagnostic yield by age in patients with only ASD indicated (569 (20.0%) females, 2281 (80.0%) males, Total *n* = 2850).

Age in Years	Number of Tests	Pathogenic (% Yield)	VOUS (% Yield)	Normal (% Yield)
0–3.4 *	701	24 (3.4%)	126 (18.0%)	551 (78.6%)
3.5–5.4	661	21 (3.2%)	119 (18.0%)	521 (78.8%)
5.5–10	768	32 (4.2%)	160 (20.8%)	576 (75.0%)
10.1–18	631	44 (7.0%)	110 (17.4)	477 (75.6%)
18+	89	5 (5.6%)	14 (15.7%)	70 (78.7%)
Total	2850	126 (4.4%)	529 (18.6%)	2195 (77.0%)

* Due to the typical age of clinical recognition and diagnoses for ASD, the range of 0–3.4 years was used for the youngest grouping in this Table.

**Table 7:** Rates of diagnostic findings on CMA by gender and diagnostic referral codes grouping.

CMA Result Type Rates by Gender	Non-ASD	ASD+	ASD only	All
**Female**				
Pathogenic	14.4%	8.5%	5.3%	11.4%
VOUS	20.9%	19.5%	17.2%	19.9%
Normal	64.7%	72.0%	77.5%	68.7%
**Male**				
Pathogenic	11.4%	5.9%	4.2%	7.5%
VOUS	19.6%	19.4%	18.9%	19.3%
Normal	69.0%	74.7%	76.9%	73.2%

## Abbreviations

CNV	copy number variant
CMA	chromosomal microarray
ASD	autism spectrum disorder
ID	intellectual disability
DD	developmental delay
MCA	multiple congenital anomalies
